# Cost–Benefit Analysis of Trans-Arterial Radio-Embolization with Y-90 Glass Microspheres Versus Drug-Eluting Bead Trans-Arterial Chemo-Embolization in Patients with Hepatocellular Carcinoma in Italy

**DOI:** 10.1007/s00270-025-04214-4

**Published:** 2025-10-06

**Authors:** Carla Rognoni, Sherry Bhoori, Laura Crocetti, Cristina Mosconi, Paolo Fonio, Marco Angelo Bongini, Elena Bozzi, Maurizia Brunetto, Alberta Cappelli, Carlo Chiesa, Roberto Cioni, Fernando Di Gregorio, Andrea Doriguzzi, Marco Maccauro, Gianluca Masi, Massimo Sponza, Carlo Spreafico, Alessandro Vit, Rosanna Tarricone

**Affiliations:** 1 Centre for Research on Health and Social Care Management (CERGAS), SDA Bocconi School of Management, Bocconi University, Milan, Italy; 2 Istituto Nazionale Tumori, Milan, Italy; 3 Azienda Ospedaliero Universitaria Pisana, Pisa, Italy; 4 Department of Radiology, IRCCS Azienda Ospedaliero Universitaria Di Bologna, Bologna, Italy; 5 Azienda Ospedaliero Universitaria Città Della Salute E Della Scienza, Turin, Italy; 6 Azienda Sanitaria Universitaria Integrata PO “S. Maria Della Misericordia”, Udine, Italy; 7 Department of Social and Political Sciences, Bocconi University, Milan, Italy

**Keywords:** Selective internal radio-embolization, Trans-arterial radio-embolization, TARE, Trans-arterial chemo-embolization, TACE, Hepatocellular carcinoma, Cost–benefit analysis, Incremental net monetary benefit, Micro-costing, Hospital perspective

## Abstract

**Purpose:**

To evaluate the cost–benefit of Trans-Arterial Radio-Embolization (TARE) with Y-90 glass microspheres compared to Drug-Eluting Bead Trans-Arterial Chemo-Embolization (DEB-TACE) in patients with intermediate- and early-stage hepatocellular carcinoma (HCC) not eligible for surgery or ablation.

**Materials and Methods:**

A partitioned survival model estimated life years (LYs) and costs over a 2-year horizon, considering the complete initial care pathway. The analysis was conducted in two scenarios, TARE with standard (SD) or personalized dosimetry (PD). Clinical data were sourced and adapted from the TRACE study, and real-world resource utilization and costs were collected from five high-volume Italian oncology centers. A micro-costing approach assessed value for money from the hospital perspective, expressed as Incremental Net Monetary Benefit (INMB), applying a willingness-to-pay (WTP) threshold of 50,000€/LY.

**Results:**

TARE showed greater survival (SD: 1.617 LYs, PD: 1.823 LYs vs 1.331 LYs DEB-TACE) and higher overall costs (SD: 32,381€, PD: 32,922€ vs 27,735€ DEB-TACE) at 2 years, reflecting its greater healthcare utilization driven by better outcomes. TARE was associated with a positive INMB (SD: 9,664€; PD: 19,429€), demonstrating cost-effectiveness.

**Conclusion:**

Due to improved survival and a positive INMB under both standard and personalized dosimetry, TARE is more cost-effective than DEB-TACE, showing greater value for money compared to DEB-TACE. These results aim to support informed decision-making on the treatment options for patients with unresectable HCC.

**Graphical Abstract:**

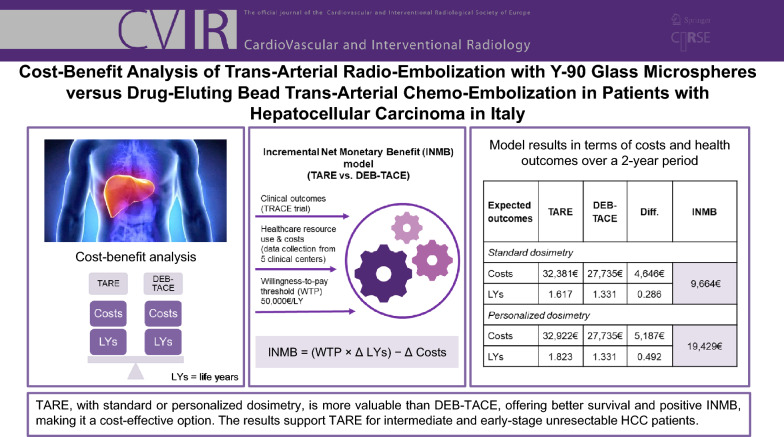

**Supplementary Information:**

The online version contains supplementary material available at 10.1007/s00270-025-04214-4.

## Introduction

Hepatocellular carcinoma (HCC) is the most common primary liver tumor and the second leading cause of cancer-related mortality worldwide. Its global burden is increasing due to rising cases of metabolic dysfunction-associated steatotic liver disease, metabolic syndrome, and chronic hepatitis B and C [1, 2]. Despite advancements in treatment, the prognosis for HCC remains poor, with a probability of living other 4 years after overcoming the first year after diagnosis of about 40% in Italy [3, 4].

As HCC typically arises in a context where liver reserve is already compromised, treatment options are often limited. In this scenario, the treatment strategy needs to be carefully personalized and may not be adherent to current guidelines due to the complex nature of the disease and the delicate balance required between effective tumor control and liver function preservation.

Locoregional therapies are applied for the full spectrum of patients who are not candidates for curative options or to downstage/downsize HCC to curative options [5]. Among the treatment options for HCC in current guidelines [5–7], Trans-Arterial Chemo-Embolization (TACE) and Trans-Arterial Radio-Embolization (TARE) with yttrium-90 (Y-90) microspheres are reported as treatment choices for intermediate substage B2 of disease (Barcelona Clinic Liver Cancer B2). TACE is the standard of care [8] and can be performed as conventional TACE (cTACE), using a chemotherapy-lipiodol emulsion followed by embolization, or as drug-eluting bead TACE (DEB-TACE), which delivers chemotherapy via micro-beads infused in the hepatic artery that also occlude tumor-feeding vessels.

TARE involves the administration of radioactive microspheres containing Y-90 directly into the hepatic artery. These microspheres lodge in the blood vessels feeding the tumor, delivering targeted beta radiation that destroys cancer cells. Personalized dosimetry provides precise, patient-specific assessment of radiation distribution, accounting for tissue and anatomical differences to maximize treatment effectiveness while minimizing harm to healthy tissue.

Emerging data show TARE may lead to superior tumor control over TACE; however, patient selection, tumor characteristics, and liver function are critical factors influencing treatment choice [3]. Compared with TACE, TARE with Y-90 glass microspheres has shown increased time to progression [9–12], longer survival [11], good quality of life [13], strong antitumoral activity in patients with portal vein invasion [14], reduced number of treatment sessions, and lower post-embolization syndrome. TARE can be used with local control or curative intent, including downstaging [15–17] in an intent-to-resect/transplant strategy [7]. In HCC, late diagnosis is a major challenge, as patients are often diagnosed beyond immediate eligibility for curative treatments like surgical resection or liver transplantation [1, 18]. For patients awaiting liver transplantation, TARE can effectively control tumor progression, reducing the risk of patients dropping out of the transplant list due to disease progression, i.e., "bridging", helping maintain tumors stable within transplant criteria. In patients whose tumors exceed transplant or resection eligibility criteria, TARE can reduce tumor size and extent, potentially making patients eligible for curative treatments, i.e., "downstaging", allowing for a transition from being ineligible to meeting the surgical criteria.

The study conducts a cost–benefit analysis from the hospital perspective in Italy, comparing the clinical pathways of patients managed with TARE with Y-90 glass microspheres versus DEB-TACE to assess the value for money for patients with intermediate-stage and early-stage HCC not eligible for surgery or ablation.

Unlike earlier analysis on the costs of these procedures [19–21], which either do not account for the clinical benefits of improved survival, relied on aggregated cost data, or do not evaluate the complete initial care pathway—including the index procedure and associated hospital resource use up to first disease progression—our study integrates survival-based outcomes with a micro-costing approach to provide a more comprehensive and precise evaluation of value.

By helping determine which treatment offers better value for money, balancing financial burden and clinical outcomes, the analysis informs value-driven decision-making, providing evidence to support the process on HTA regulation at the European level [22, 23] and the Italian program of HTA for medical devices [24].

## Materials and Methods

TRACE, a recent phase II randomized controlled trial (RCT) comparing TARE with Y-90 glass microspheres (TheraSphere, Boston Scientific) versus DEB-TACE (DC Bead, Boston Scientific), has been used as a source of clinical data for the evaluation [11]. Participants had intermediate-stage HCC, extended to Eastern Cooperative Oncology Group performance status 1, and early-stage HCC not eligible for surgery or ablation.

A micro-costing analysis was conducted to estimate hospital costs associated with a defined segment of the care pathway, encompassing first-line treatment (TARE or DEB-TACE), complication management, follow-up visits, imaging, laboratory testing, and the first subsequent treatment. Micro-costing is a precise cost analysis method that identifies, measures, and values all components of a healthcare service to determine its true cost. It provides highly accurate estimates, making it useful for comparing interventions, especially when standard tariffs like Diagnosis-Related Groups (DRGs) may underestimate costs for complex procedures.

The analysis has been reported according to the Consolidated Health Economic Evaluation Reporting Standards (CHEERS) [25, 26].

### The Model

**Fig. 1:**
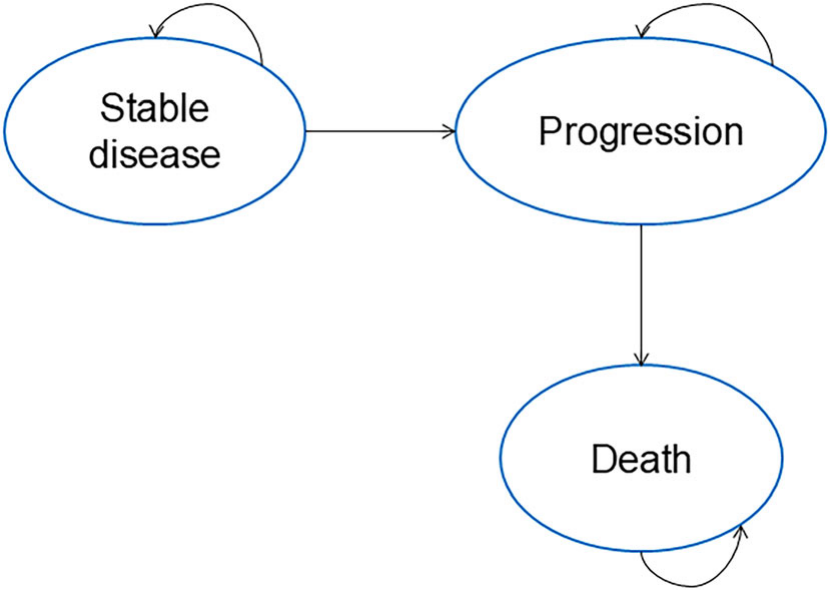
Representation of the implemented model

A partition survival model with “stable disease,” “progression,” and “death” health states has been developed to estimate the costs and life expectancy (life years—LYs) associated with TARE and DEB-TACE (Fig. 1). The population considered in the model comprised adults with a mean age of 67.5 years (87.5% males), according to the population analyzed by TRACE [11]. This population reflects the indications of national [27] (Italy) and international guidelines [28, 29] for the management of HCC with the two treatments considered.

All patients begin the process in the “stable disease” state (responder patients). If the disease progresses (non-responder patients), they move to the “progression” state and cannot revert to an earlier state. The model operates with a 1-month cycle length. This partition survival model, frequently used in appraisals, directly estimates state occupancy from survival data. The distribution of patients across health states over time is derived from overall survival (OS) and progression-free survival (PFS) data from the TRACE trial [11]. The model spans a 2-year time horizon to cover the patients’ pathway. Beyond this period, other factors may influence patient care, making it harder to isolate the true value of the evaluated treatments.

Clinical and economic outcomes are projected using curve fittings. For DEB-TACE, various functions were used to fit parameters for OS and PFS, with the most plausible models selected statistically (see Appendix for details). Hazard ratios (HRs) for TARE (HR_OS_ = 0.490, HR_PFS_ = 0.360) from the trial were applied to DEB-TACE curves to create curves for TARE. LYs for TARE and DEB-TACE were estimated through the model using the obtained OS curves (by calculating for each the area under the survival curve over the defined time horizon). These two curves represent the probability of survival over time for patients treated with TARE with Y-90 glass microspheres and DEB-TACE, respectively. The model calculations ensure that, regardless of the parametric distribution used to extrapolate the survival curves, OS is always greater than or equal to PFS. Since mortality for HCC is, in general, higher than background mortality rates for the Italian population [30], no adjustments for overall mortality have been made.

### Healthcare Resource Utilization and Micro-Costing Analysis

Data on the frequency of healthcare resource use and their associated costs were collected through a questionnaire administered in July 2024 to clinicians in five high-volume clinical centers managing patients with HCC, geographically distributed across Italy.^1^ The questionnaire included different sections:

1. Description of patients’ characteristics and the locoregional procedures
2. Healthcare services, including visits, examinations, and imaging during the peri-procedural period (first month) and follow-up, along with associated hospital costs for responders and non-responders.
3. Treatment costs include materials, healthcare personnel time, operative room use and hospitalization. For TARE, both dosimetry simulation and treatment were considered.
4. Costs for the pharmacological treatments administered before and after TARE or DEB-TACE.
5. Costs for the management of severe (grade 3/4) adverse events caused by TARE or DEB-TACE according to the list observed during the TRACE trial [11].
6. Following treatments performed in the follow-up and their costs. The mean value for each cost item was calculated from the completed questionnaires. Table 1 reports the main model parameters (€, 2024). Supplementary Table 1 reports the healthcare resource utilization. Considering that costs vary among hospitals, scenario analyses have been performed considering the minimum and maximum input parameters provided.

**Table 1:** Main model input parameters

Parameter	Baseline value	Min	Max	Reference
Mean body weight (kg)	78.25	62.60	93.90	[31]
*Hazard ratios (TARE vs. DEB-TACE)*				
HR OS	0.49	0.28	0.87	Dhondt 2022 [11]
HR PFS	0.36	0.18	0.70	
*Healthcare personnel cost (per minute)*				
Physician	1.10€	0.88€	1.32€	MEF (Ministero dell’Economia e delle Finanze—Italian Ministry of Economy and Finance) 2022 data uplifted to 2024 [32]
Nurse	0.46€	0.36€	0.55€	
Technician	0.45€	0.36€	0.54€	
Nuclear physician	1.10€	0.88€	1.32€	
Radiotherapist	1.10€	0.88€	1.32€	
*Treatment costs*				
N. TARE sessions	1.52	1.21	1.82	Dhondt 2022 [11]
N. DEB-TACE sessions	2.94	2.36	3.53	Dhondt 2022 [11]
Cost TARE	14,258.35€	11,715.13€	17,151.36€	Calculated from data provided by HCPs
Cost DEB-TACE	3,330.55€	2,162.98€	5,417.28€	Calculated from data provided by HCPs
Cost TARE (second line treatment)	14,278.55€	14,188.87€	14,408.01€	Calculated from data provided by HCPs
Cost Deb-TACE (second line treatment)	3,359.43€	2,162.98€	5,417.28€	Calculated from data provided by HCPs
Cost cTACE (second line treatment)	2,733.03€	1,179.98€	6,066.14€	Calculated from data provided by HCPs
Cost RFA (second line treatment)	1,889.11€	1,124.11€	2,659.11€	Calculated from data provided by HCPs
Cost systemic chemotherapy (second line treatment)	71,261.48€	57,009.18€	85,513.77€	Calculated from data provided by HCPs and official prices [33, 34]
Cost pharmacological treatments TARE	15.69€	0.00€	47.07€	Calculated from data provided by HCPs
Cost pharmacological treatments DEB-TACE	27.08€	10.41€	47.07€	Calculated from data provided by HCPs
*Healthcare resources costs*				
Specialist visit	48.52€	17.00€	125.00€	Calculated from data provided by HCPs
Electrocardiogram	60.00€	60.00€	60.00€	
Blood tests	33.18€	13.53€	43.50€	
Computed tomography of the abdomen	128.36€	78.00€	210.00€	
Computed tomography of the complete abdomen	169.80€	87.52€	248.90€	
Magnetic resonance imaging of the abdomen	252.63€	210.00€	286.00€	
Hepatic ultrasound	44.15€	27.00€	60.45€	
Positron emission tomography	1,100.00€	570.00€	1,530.00€	
Macro aggregated albumin scintigraphy	159.00€	70.00€	207.00€	
Arteriography	203.31€	141.45€	265.17€	
Gastroscopy	250.00€	250.00€	250.00€	
Single photon emission computed tomography	227.00€	70.00€	384.00€	
Esophago-gastro-duodenoscopy	250.00€	250.00€	250.00€	
*Costs for the management of adverse events*				
Cost blood and lymphatic system disorders	3,026.84€	2,421.47€	3,632.20€	Calculated from data provided by HCPs
Cost musculoskeletal and connective tissue disorders	1,745.00€	1,396.00€	2,094.00€	DRG 247
Cost nervous system disorders	3,026.84€	2,421.47€	3,632.20€	Calculated from data provided by HCPs
Cost cardiac disorders	1,674.50€	1,339.60€	2,009.40€	DRG 138, DRG 139 (mean)
Cost renal and urinary disorders	1,297.22€	1,037.77€	1,556.66€	Calculated from data provided by HCPs
Cost hepatobiliary disorders	4,375.47€	3,500.38€	5,250.56€	Calculated from data provided by HCPs
Cost respiratory, thoracic, and mediastinal disorders	2,133.00€	1,706.40€	2,559.60€	DRG 99, DRG 100 (mean)

HR = hazard ratio, cTACE = conventional TACE, RFA = radiofrequency ablation, HCPs = health care professionals, DRG = Diagnosis-Related Group

As to the costs of healthcare personnel involved in the procedures, we referenced salary data from the Italian Ministry of Economics and Finance database [32].

The mean number of treatment sessions per patient has been estimated by the TRACE trial [11], equal to 1.52 for TARE and 2.94 for DEB-TACE, in line with indications provided by the clinicians involved in the study and consistent with literature data [35–39].

The pharmacological treatments administered to patients in the peri-procedural period were mainly antibiotics, anti-inflammatory drugs, antiemetics, and gastric protectors, for a mean cost per patient of 15.69€ and 27.08€ for TARE and DEB-TACE, respectively. The drug costs were obtained from the Italian "Farmadati" database [29]. Regarding the management of adverse events, in the very limited cases of missing data due to difficulties in estimating the hospital cost, we referred to DRG reimbursement rates.

Subsequent treatments were recognized from the TRACE trial [11] and combined in the model. Systemic treatments performed in the clinical practice of centers involved in the study were: sorafenib, atezolizumab + bevacizumab, lenvatinib, tremelimumab + durvalumab, regorafenib, and cabozantinib. Regarding the cost of the different systemic treatments, we referred to published Italian prices [34] (Supplementary Table 2). For treatment dosages depending on the weight of the patient, we applied mean body weights for males and females for Italy [31].

It was assumed that subsequent treatments were performed at median time to progression [11], which was 9.5 months for DEB-TACE and 17.1 months for TARE. This implies that, on average, DEB-TACE patients received their first subsequent treatment within the first year, while TARE patients received it during the second year.

The analysis included only the first subsequent treatments to ensure standardization and comparability, as additional treatments vary among patients due to clinical factors. This approach allows for a focused assessment of costs directly related to the evaluated interventions.

While the baseline scenario considered standard dosimetry for the execution of TARE, a scenario analysis has been conducted considering a personalized dosimetry approach based on the evidence presented in the literature. Hazard ratios were applied for personalized dosimetry according to the trial by Garin and colleagues [40], translating into final hazard ratios of 0.21 for OS and 0.26 for PFS comparing TARE with personalized dosimetry versus DEB-TACE. In this scenario, specific adverse events reported in the cited study were considered for personalized dosimetry. No additional cost was incurred for the personalized dosimetry approach, since the cost of the software is bundled with the TARE device.

Although our analysis was primarily conducted from the Italian hospital perspective, reflecting country-specific cost structures and pricing assumptions, ensuring broader European relevance is critical, particularly in the evaluation of life-extending technologies. To test the transferability of our findings across European healthcare systems, we performed a supplemental scenario analysis in which key cost parameters were systematically varied to reflect plausible inter-country pricing differences commonly observed across European hospital systems. This approach was guided by OECD reference price level for health goods and services (indexed to 100), where Italy corresponds to a value of 96, while the Czech Republic and Switzerland represent the lower and upper extremes in Europe, with values of 33 and 172, respectively [41]. Based on this range and taking Italy as the baseline, we applied cost variations ranging from − 66% to + 79% to reflect the range of healthcare cost variability observed across European countries. This scenario analysis enhances the external validity of our model and supports the generalizability of our conclusions to diverse European healthcare contexts.

### Analyses

The TARE (standard and personalized dosimetry approaches) and DEB-TACE strategies were compared using the incremental net monetary benefit (INMB) metric. INMB is determined by calculating the difference between the monetary valuation of health benefits and the associated costs of each strategy, expressed in monetary terms, allowing direct comparison between different strategies. The valuation of benefits is typically estimated by applying a willingness-to-pay (WTP) threshold, representing the maximum amount a decision-maker is willing to pay per unit of health gain. In this analysis, a threshold of 50,000€ per life year (LY) was employed, consistent with values commonly reported in the Italian literature for health economic evaluations [42–44].

A positive INMB indicates that TARE is a cost-effective strategy relative to DEB-TACE at the specified WTP threshold, meaning that the cost of obtaining the health benefit remains within acceptable limits from a decision-maker’s perspective. This methodological approach aligns with established principles of economic evaluation and is a cornerstone of the evidence-informed, value-based healthcare paradigm [45–49].

## Results

Table 2 shows the detailed cumulative costs for each treatment strategy over a time horizon of 2 years. In the base-case analysis, where TARE was performed with standard dosimetry, the total cost per patient was 32,381€ for TARE compared to 27,735€ for DEB-TACE, resulting in a cost difference of 4,646€. The personalized dosimetry scenario showed a slight increase in total cost (32,922€), mainly related to additional visits/exams due to a higher survival rate. Costs reflect longer survival and better response with TARE, meaning more patients remain under follow-up and overall costs are distributed across a larger patient population, resulting in higher cumulative resource use.

**Table 2:** Economic impact of TARE compared to DEB-TACE on the clinical pathway over a 2-year period

Model health state	Healthcare resources	Cost of healthcare resources (€)		Difference (€)
		TARE	DEB-TACE	
*Standard dosimetry scenario*				
Progression-free	Initial treatment	21,604	9,807	11,797
	Visits/exams	4,295	2,173	2,121
	Pharmacological treatments	16	27	− 11
	Management of adverse events	2,053	2,352	− 299
Progression	Visits/exams	2,053	1,346	893
	Subsequent treatments	2,205	12,030	− 9,825
TOTAL		32,381	27,735	4,646
*Personalized dosimetry scenario*				
Progression-free	Initial treatment	21,604	9,807	11,797
Progression-free	Visits/exams	4,501	2,173	2,328
	Pharmacological treatments	16	27	− 11
	Management of adverse events	1,961	2,352	− 391
Progression	Visits/exams	2,635	1,346	1,289
	Subsequent treatments	2,205	12,030	− 9,825
TOTAL		32,922	27,735	5,187

**Fig. 2:**
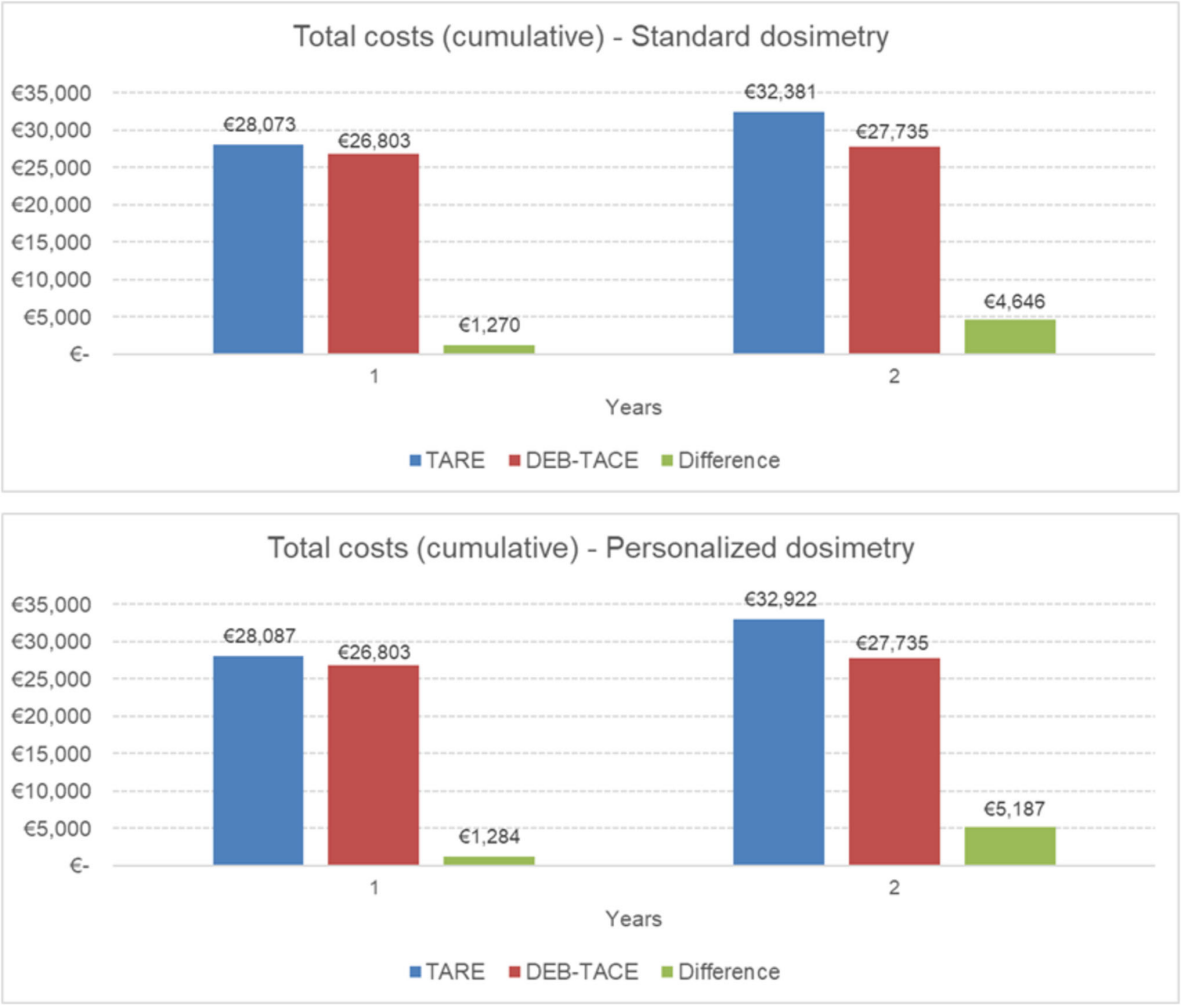
Details on overall costs

Adverse events management costs, lower in the standard dosimetry scenario, improve further with personalized dosimetry. Detailed overall costs are reported in Fig. 2. The difference in subsequent treatment costs between TARE and DEB-TACE is explained by the timing of retreatment. Based on median progression times, DEB-TACE patients typically required retreatment in year 1, while TARE patients did so in year 2. The analysis included only the first retreatment, and no additional treatments were accounted for beyond that point for either strategy. It is therefore not excluded that DEB-TACE patients may have required further interventions in year 2; however, these were not captured, as the methodology consistently counted only the first retreatment per patient.

Table 3 presents the results of the comparative cost–benefit analysis at 2 years.

**Table 3:** Summary of model results in terms of costs and health outcomes over a 2-year period

Expected outcomes	TARE	DEB-TACE	Difference	Incremental Net Monetary Benefit (INMB)
*Standard dosimetry*				
Costs	32,381€	27,735€	4,646€	9,664€
LYs	1.617	1.331	0.286	
*Personalized dosimetry*				
Costs	32,922€	27,735€	5,187€	19,429€
LYs	1.823	1.331	0.492	

TARE with standard dosimetry (SD) provides 1.617 life years, 0.29 years more than DEB-TACE (1.331 LYs). The incremental net monetary benefit is positive (+ 9,664€), demonstrating that TARE with SD provides greater value for money than DEB-TACE. A personalized dosimetry (PD) approach enables patients to have an even better cost-effectiveness profile, with its increased survival advantage (1.823 vs. 1.331 LYs). The INMB is positive, amounting to + 19,429€, proving that TARE with PD is cost-effective and provides greater value than DEB-TACE, making it the preferred option.^2^ If we divide the total cost per the LYs lived, we see how it reflects the INMB, with a difference between TARE and DEB-TACE in 2 years of − 816€ for SD and − 2,784€ for PD (SD: 20,027€/LY, PD: 18,059€/LY vs. DEB-TACE: 20,843€/LY) – meaning that TARE has a lower cost per unit of survival. The scenario analysis performed considering minimum input data led to costs for TARE and DEB-TACE strategies of 19,240€ and 14,494€, respectively, with a cost difference of 4,746€. LYs in this setting were 1.766 and 1.331, respectively, with an INMB of 16,999€. The maximum input parameters showed costs for TARE and DEB-TACE pathways of 48,451€ and 52,700€, respectively, with a cost difference of 4,249€ in favor of TARE. LYs in this scenario were 1.395 and 1.331, respectively, with an INMB of 7,465€.

Scenario analyses simulating the lower and upper bounds of healthcare cost variability across European settings resulted in INMBs of 20,065€ and 1,134€, respectively. These findings demonstrate the robustness of the model and indicate that TARE remains a cost-effective strategy across a wide range of cost assumptions applicable to diverse European healthcare systems.

## Discussion

Locoregional treatments are pivotal in the multidisciplinary management of HCC, providing effective options for disease control, particularly in patients who are not candidates for surgery. Economic evaluations often focus only on procedural costs, overlooking the full clinical pathway and the costs that may occur following the care episode. A comprehensive approach is essential, especially for providers managing complex conditions like unresectable HCC, where multiple treatments may be required. Higher upfront costs may ultimately be more cost-effective due to improved patient outcomes.

In the present study, we assessed the costs from the hospital perspective for managing patients with HCC, trying to overcome the limits of current available economic literature, likely underestimating costs due to unaccounted hospitalizations and side effects management [18]. The findings of this study align with existing literature on the cost-effectiveness of TARE in different healthcare systems [21, 50, 51] and on the higher value of PD vs. SD [52].

The analyses showed for TARE a positive INMB for both standard (INMB = 9664€) and personalized dosimetry (INMB = 19,429€) compared to DEB-TACE. Personalized dosimetry leads to a meaningful improvement in the objective response rate and overall survival [40] compared to standard dosimetry; aligned with this, the model outcomes improve further when considering a scenario that involves personalized dosimetry. The study emphasizes that the initially higher cost of TARE is justified by the additional survival benefit it provides and can therefore be considered an investment that yields greater long-term benefits for patients; this makes TARE a preferable choice from a cost-effectiveness perspective.

TARE represents a key therapeutic option for selected patients with unresectable HCC [53], offering a clear advantage in downstaging to curative treatments such as liver transplantation, as demonstrated by higher success rates compared to DEB-TACE in the TRACE study [11]. This approach supports both curative intent and local tumor control, contributing to continuous improvements in patient management. This highlights its role in enhancing patient outcomes and aligns with the principles of value-based healthcare [46].

Although the analysis was conducted from an Italian healthcare perspective, the inclusion of a scenario analysis incorporating inter-country pricing variability—based on OECD reference indices for health services—provides a robust approximation of cost structures across a wide range of European healthcare systems. By simulating healthcare cost levels from the lowest (e.g., Czech Republic) to the highest (e.g., Switzerland), the model demonstrated consistently positive INMB values. This confirms the robustness of our findings and supports their applicability to diverse national contexts, suggesting that TARE is likely to remain a cost-effective option throughout the European region. Future research should aim to refine the analysis through collaboration with national HTA agencies and the integration of real-world data from diverse healthcare settings.

The present study has some limitations that must be acknowledged. The clinical effectiveness of TARE and DEB-TACE was derived from a recently published phase II RCT [11], which demonstrated superior tumor control and survival with yttrium-90 glass radio-embolization compared to DEB-TACE in selected patients with unresectable HCC. Later, two studies on smaller populations showed a consistent trend of lower recurrence, longer time to progression, and survival [12, 54]. The use of data from a phase II study represents a limitation, particularly given the small sample size and the exploratory nature of the trial. However, in the absence of large phase III randomized trials directly comparing TARE and DEB-TACE, the available phase II evidence, while preliminary, provides the most robust comparative data currently accessible for modeling purposes. However, caution should anyhow be exercised when generalizing the results to a broader population, as confirmation from larger, adequately powered randomized trials is necessary before drawing definitive conclusions.

Another limitation relates to the exclusion from the analysis of the bridging effect of TARE for liver transplantation, as the TRACE trial did not report separate PFS curves for post-transplant outcomes, and it was not possible to isolate the impact of transplantation on progression-free survival. Moreover, the relatively short trial duration may not have been sufficient to capture the long-term benefits associated with transplantation.

Cost data for the micro-costing analysis were collected through structured questionnaires, referencing hospital records and budget systems. When direct figures were unavailable, estimates were aligned with administrative data for consistency. Patient-level cost tracking would ensure the highest precision, but the lack of standardized methods for tracking costs across the patient care pathway and challenges in accessing detailed administrative data limit this possibility. On the other hand, a micro-costing approach allows for overcoming limitations that may be posed by DRGs' approximation, often underrepresenting actual resource use. Moreover, the analysis focused only on the first recurrence event and did not account for subsequent lines of treatment or long-term follow-up, which may underestimate the overall clinical and economic impact of disease progression over time.

Evaluations on the medical devices considered in this study present particular challenges due to rapid innovation, outcomes influenced by user training and competence (learning curve), and dynamic pricing [22, 55]. Centers that perform a higher volume of procedures may achieve improved health outcomes and reduced procedure costs [55].

While the present analysis was conducted from the hospital perspective and included only direct healthcare costs, future research should adopt a broader societal perspective to capture indirect costs such as productivity losses, caregiver burden, and out-of-pocket expenses, which may significantly influence the overall value of the intervention.

## Conclusion

Our findings indicate that TARE offers greater value for money than DEB-TACE for patients with intermediate or early-stage HCC not eligible for surgery, ablation, or transplant. TARE improves survival, reduces adverse events, and has a positive incremental net monetary benefit, making it cost-effective at a willingness-to-pay threshold of 50,000€/LY. These findings are consistent with existing cost-effectiveness evidence and can support both clinicians and healthcare administrators in informed decision-making. Future research should further investigate the broader system-level impact of TARE, building on the current body of evidence.

## Supplementary Information

Below is the link to the electronic supplementary material.

Supplementary file1 (DOCX 29 KB)

Supplementary file2 (DOCX 15 KB)

Supplementary file3 (DOCX 81 KB)

## Footnotes

1 Azienda Ospedaliero Universitaria Città della Salute e della Scienza, Torino; Istituto Nazionale Tumori, Milano; Azienda Sanitaria Universitaria Integrata PO “S. Maria della Misericordia”, Udine; IRCCS Azienda Ospedaliero Universitaria di Bologna, Bologna; Azienda Ospedaliero Universitaria Pisana, Pisa.

2 A higher INMB shows greater value for money, reflecting the more favorable balance between costs and health outcomes of personalized dosimetry compared to standard dosimetry.
