# Supplementary material for: Cost–Benefit Analysis of Trans-Arterial Radio-Embolization with Y-90 Glass Microspheres Versus Drug-Eluting Bead Trans-Arterial Chemo-Embolization in Patients with Hepatocellular Carcinoma in Italy
Source: Cardiovasc Intervent Radiol. 2025 Oct 6;48(11):1614–24. doi: 10.1007/s00270-025-04214-4 (PMC12572073; doi:10.1007/s00270-025-04214-4)
Supplement: Supplementary file 1 — Supplementary file1 (DOCX 29 KB) [file 270_2025_4214_MOESM1_ESM.docx]

Supplemetary Table 1 – Healthcare resource use

| **Parameter** | **Baseline value** | **Min** | **Max** | **Reference** |
| --- | --- | --- | --- | --- |
| **Second line treatments (% of patients)** | | | | |
| % DEB-TACE after TARE | 6.25% | 5.0% | 7.5% | Dhondt 2022 [11] |
| % cTACE after TARE | 21.88% | 17.5% | 26.3% | Dhondt 2022 [11] |
| % TARE after TARE | 9.38% | 7.5% | 11.3% | Dhondt 2022 [11] |
| % RFA after TARE | 3.13% | 2.5% | 3.8% | Dhondt 2022 [11] |
| % DEB-TACE after DEB-TACE | 26.47% | 21.2% | 31.8% | Dhondt 2022 [11] |
| % cTACE after DEB-TACE | 8.82% | 7.1% | 10.6% | Dhondt 2022 [11] |
| % TARE after DEB-TACE | 2.94% | 2.4% | 3.5% | Dhondt 2022 [11] |
| % Chemotherapy after DEB-TACE | 14.71% | 11.8% | 17.6% | Dhondt 2022 [11] |
| **Adverse events (% of patients)** | | | | |
| **TARE** | | | | |
| Renal and urinary disorders | 15.2% | 12.1% | 18.2% | Dhondt 2022 [11] |
| Hepatobiliary disorders | 42.4% | 33.9% | 50.9% | Dhondt 2022 [11] |
| **DEB-TACE** | | | | |
| Blood and lymphatic system disorders | 2.8% | 2.2% | 3.3% | Dhondt 2022 [11] |
| Musculoskeletal and connective tissue disorders | 5.6% | 4.4% | 6.7% | Dhondt 2022 [11] |
| Nervous system disorders | 2.8% | 2.2% | 3.3% | Dhondt 2022 [11] |
| Cardiac disorders | 5.6% | 4.4% | 6.7% | Dhondt 2022 [11] |
| Renal and urinary disorders | 13.9% | 11.1% | 16.7% | Dhondt 2022 [11] |
| Hepatobiliary disorders | 33.3% | 26.7% | 40.0% | Dhondt 2022 [11] |
| Respiratory, thoracic, and mediastinal disorders | 16.7% | 13.3% | 20.0% | Dhondt 2022 [11] |
| **Healthcare resource use frequencies** | | | | |
| **Peri-procedural period** | | | | |
| TARE specialist visit | 2.00 | 2.00 | 2.00 | Calculated from data provided by HCPs |
| TARE electrocardiogram | 0.03 | 0.00 | 0.80 | Calculated from data provided by HCPs |
| TARE blood tests | 2.00 | 2.00 | 2.00 | Calculated from data provided by HCPs |
| TARE computed tomography of the abdomen | 1.17 | 0.30 | 2.00 | Calculated from data provided by HCPs |
| TARE computed tomography of the complete abdomen | 1.88 | 1.40 | 2.00 | Calculated from data provided by HCPs |
| TARE magnetic resonance imaging of the abdomen | 0.30 | 0.00 | 0.60 | Calculated from data provided by HCPs |
| TARE hepatic ultrasound | 0.83 | 0.50 | 1.00 | Calculated from data provided by HCPs |
| TARE positron emission tomography | 1.00 | 1.00 | 1.00 | Calculated from data provided by HCPs |
| TARE macro aggregated albumin scintigraphy | 1.00 | 1.00 | 1.00 | Calculated from data provided by HCPs |
| TARE arteriography | 2.00 | 2.00 | 2.00 | Calculated from data provided by HCPs |
| TARE gastroscopy | 0.37 | 0.00 | 1.00 | Calculated from data provided by HCPs |
| TARE single photon emission computed tomography | 1.00 | 1.00 | 1.00 | Calculated from data provided by HCPs |
| DEB-TACE specialist visit | 2.00 | 2.00 | 2.00 | Calculated from data provided by HCPs |
| DEB-TACE electrocardiogram | 0.09 | 0.00 | 0.80 | Calculated from data provided by HCPs |
| DEB-TACE blood tests | 2.00 | 2.00 | 2.00 | Calculated from data provided by HCPs |
| DEB-TACE computed tomography of the abdomen | 0.86 | 0.00 | 2.00 | Calculated from data provided by HCPs |
| DEB-TACE computed tomography of the complete abdomen | 1.57 | 0.70 | 2.00 | Calculated from data provided by HCPs |
| DEB-TACE magnetic resonance imaging of the abdomen | 0.15 | 0.00 | 0.30 | Calculated from data provided by HCPs |
| DEB-TACE ultrasound abdomen | 1.25 | 0.50 | 2.00 | Calculated from data provided by HCPs |
| DEB-TACE positron emission tomography | 0.00 | 0.00 | 0.00 | Calculated from data provided by HCPs |
| DEB-TACE esophago-gastro-duodenoscopy | 0.01 | 0.00 | 0.05 | Calculated from data provided by HCPs |
| **Progression (diagnostic phase)** | | | | |
| TARE visit | 2.00 | 2.00 | 2.00 | Calculated from data provided by HCPs |
| TARE electrocardiogram | 0.00 | 0.00 | 0.00 | Calculated from data provided by HCPs |
| TARE blood tests | 2.00 | 2.00 | 2.00 | Calculated from data provided by HCPs |
| TARE computed tomography of the abdomen | 1.50 | 1.00 | 2.00 | Calculated from data provided by HCPs |
| TARE computed tomography of the complete abdomen | 1.88 | 1.40 | 2.00 | Calculated from data provided by HCPs |
| TARE magnetic resonance imaging of the abdomen | 0.38 | 0.30 | 0.45 | Calculated from data provided by HCPs |
| TARE hepatic ultrasound | 0.83 | 0.50 | 1.00 | Calculated from data provided by HCPs |
| TARE positron emission tomography | 1.00 | 1.00 | 1.00 | Calculated from data provided by HCPs |
| TARE macro aggregated albumin scintigraphy | 1.00 | 1.00 | 1.00 | Calculated from data provided by HCPs |
| TARE arteriography | 2.00 | 2.00 | 2.00 | Calculated from data provided by HCPs |
| TARE gastroscopy | 0.50 | 0.00 | 1.00 | Calculated from data provided by HCPs |
| TARE single photon emission computed tomography | 1.00 | 1.00 | 1.00 | Calculated from data provided by HCPs |
| DEB-TACE visit | 2.00 | 2.00 | 2.00 | Calculated from data provided by HCPs |
| DEB-TACE electrocardiogram | 0.00 | 0.00 | 0.00 | Calculated from data provided by HCPs |
| DEB-TACE blood tests | 2.00 | 2.00 | 2.00 | Calculated from data provided by HCPs |
| DEB-TACE computed tomography of the abdomen | 1.50 | 1.00 | 2.00 | Calculated from data provided by HCPs |
| DEB-TACE computed tomography of the complete abdomen | 1.85 | 1.40 | 2.00 | Calculated from data provided by HCPs |
| DEB-TACE magnetic resonance imaging of the abdomen | 0.33 | 0.20 | 0.45 | Calculated from data provided by HCPs |
| DEB-TACE ultrasound abdomen | 1.25 | 0.50 | 2.00 | Calculated from data provided by HCPs |
| DEB-TACE positron emission tomography | 0.05 | 0.05 | 0.05 | Calculated from data provided by HCPs |
| **Healthcare resource use frequencies (per model cycle=1 month)** | | | | |
| **PFS** | | | | |
| TARE specialist visit | 0.28 | 0.17 | 0.33 | Calculated from data provided by HCPs |
| TARE blood tests | 0.28 | 0.17 | 0.33 | Calculated from data provided by HCPs |
| TARE computed tomography of the abdomen | 0.18 | 0.03 | 0.33 | Calculated from data provided by HCPs |
| TARE computed tomography of the complete abdomen | 0.28 | 0.12 | 0.33 | Calculated from data provided by HCPs |
| TARE magnetic resonance imaging of the abdomen | 0.05 | 0.00 | 0.10 | Calculated from data provided by HCPs |
| TARE hepatic ultrasound | 0.28 | 0.17 | 0.33 | Calculated from data provided by HCPs |
| DEB-TACE specialist visit | 0.46 | 0.17 | 1.26 | Calculated from data provided by HCPs |
| DEB-TACE blood tests | 0.42 | 0.17 | 1.05 | Calculated from data provided by HCPs |
| DEB-TACE computed tomography of the abdomen | 0.28 | 0.00 | 1.05 | Calculated from data provided by HCPs |
| DEB-TACE computed tomography of the complete abdomen | 0.43 | 0.12 | 1.05 | Calculated from data provided by HCPs |
| DEB-TACE magnetic resonance imaging of the abdomen | 0.09 | 0.00 | 0.32 | Calculated from data provided by HCPs |
| DEB-TACE ultrasound abdomen | 0.28 | 0.17 | 0.33 | Calculated from data provided by HCPs |
| **Progression** | | | | |
| TARE specialist visit | 0.37 | 0.33 | 0.50 | Calculated from data provided by HCPs |
| TARE blood tests | 0.37 | 0.33 | 0.50 | Calculated from data provided by HCPs |
| TARE computed tomography of the abdomen | 0.27 | 0.08 | 0.33 | Calculated from data provided by HCPs |
| TARE computed tomography of the complete abdomen | 0.31 | 0.23 | 0.33 | Calculated from data provided by HCPs |
| TARE magnetic resonance imaging of the abdomen | 0.10 | 0.10 | 0.10 | Calculated from data provided by HCPs |
| TARE hepatic ultrasound | 0.28 | 0.17 | 0.33 | Calculated from data provided by HCPs |
| DEB-TACE specialist visit | 0.46 | 0.33 | 0.67 | Calculated from data provided by HCPs |
| DEB-TACE blood tests | 0.46 | 0.33 | 0.67 | Calculated from data provided by HCPs |
| DEB-TACE computed tomography of the abdomen | 0.25 | 0.08 | 0.33 | Calculated from data provided by HCPs |
| DEB-TACE computed tomography of the complete abdomen | 0.33 | 0.23 | 0.42 | Calculated from data provided by HCPs |
| DEB-TACE magnetic resonance imaging of the abdomen | 0.09 | 0.07 | 0.10 | Calculated from data provided by HCPs |
| DEB-TACE ultrasound abdomen | 0.28 | 0.17 | 0.33 | Calculated from data provided by HCPs |

PFS=progression-free survival, HCPs=health care professionals
