# Supplementary material for: Cost–Benefit Analysis of Trans-Arterial Radio-Embolization with Y-90 Glass Microspheres Versus Drug-Eluting Bead Trans-Arterial Chemo-Embolization in Patients with Hepatocellular Carcinoma in Italy
Source: Cardiovasc Intervent Radiol. 2025 Oct 6;48(11):1614–24. doi: 10.1007/s00270-025-04214-4 (PMC12572073; doi:10.1007/s00270-025-04214-4)
Supplement: Supplementary file 2 — Supplementary file2 (DOCX 15 KB) [file 270_2025_4214_MOESM2_ESM.docx]

| **Treatment** | **Mean treatment duration (months)** | **Treatment distribution** | **Cost** |
| --- | --- | --- | --- |
| Sorafenib | 5.3 | 14% | 11,509 € |
| Atezolizumab+Bevacizumab | 7.8 | 66% | 81,512 € |
| Lenvatinib | 10.3 | 18% | 37,572 € |
| STRIDE (Tremelimumab+Durvalumab) | 3.4 | 20% | 26,226 € |
| Regorafenib | 4 | 5% | 7,545 € |
| Cabozantinib | 6 | 10% | 35,412 € |
| TOTAL |  |  | 71,261 € |

Supplementary Table 2 – Costs for systemic treatments
